# Transabdominal Renal Doppler Ultrasound in Healthy Adult Holstein-Friesian Cows: A Pilot Study

**DOI:** 10.3390/ani11010063

**Published:** 2020-12-31

**Authors:** J. Daniel Barreiro-Vázquez, Marta Miranda, Andrés Barreiro-Lois

**Affiliations:** 1 Department of Anatomy, Animal Production and Clinical Veterinary Sciences, Faculty of Veterinary Medicine, University of Santiago de Compostela, 27002 Lugo, Spain; josedaniel.barreiro@usc.es (J.D.B.-V.); andres.barreiro@usc.es (A.B.-L.); 2 Veterinary Teaching Hospital “Rof-Codina”, Faculty of Veterinary Medicine, University of Santiago de Compostela, 27002 Lugo, Spain

**Keywords:** Doppler ultrasound, cow, kidney, resistive index, pulsatility index

## Abstract

**Simple Summary:**

Knowledge of the physiological renal blood flow in cattle is essential for interpretation of Doppler ultrasound. In this paper, we describe a protocol for obtaining the renal Doppler parameters resistive index (RI) and pulsatility index (PI) in a systematic ultrasound evaluation of the kidney in cattle and provide preliminary reference values for healthy adult Holstein-Friesian cows.

**Abstract:**

There is a notable lack of reference values for the renal resistive indices in the bovine kidney. Ultrasound (US) Doppler evaluation of these indices is a powerful, non-invasive technique for assessing, monitoring and diagnosing renal diseases in humans and other animals (e.g., small animals and horses). The aims of the present study were to establish a protocol for renal Doppler US in adult healthy Holstein-Friesian cows and to provide reference values for the renal resistive index (RI) and pulsatility index (PI). In cattle, the right kidney is always visible through a right abdominal window. Nevertheless, the left kidney is rarely accessible by transabdominal ultrasound. Doppler evaluation of the kidneys via a transabdominal approach is possible when accessible, but measurements can only be made in the larger vessels at the renal hilum. Normal RI and PI values were respectively 0.49 ± 0.07 and 0.70 ± 0.15 for the right kidney and 0.53 ± 0.05 and 0.79 ± 0.11 for the left kidney. We suggest an upper cut-off value for the RI of 0.63 and for the PI of 1.00 in healthy Holstein-Friesian cows. This is the first report describing normal values for the renal RI and PI in cattle that may be useful in future studies for characterizing different bovine pathologies that affect the renal parenchyma.

## 1. Introduction

Ultrasound (US) evaluation of the kidneys is a routine, non-invasive technique used in both human and veterinary practice. B-mode US helps bovine clinicians to distinguish between upper and lower urinary tract infection, focal or diffuse renal involvement, and sometimes between acute and chronic kidney disease [1,2,3,4,5,6,7,8]. However, it cannot differentiate many kidney diseases, especially diffuse diseases, i.e., toxicosis and inflammatory or infectious diseases [6,7], because of its low specificity in these situations [9,10]. Transcutaneous renal B-mode US examination in cattle is well documented, indicating that only the right kidney is usually visible in normal conditions and difficult to measure because the entire organ is not completely included in one view [1,2,7]. A transabdominal ultrasound protocol involving an assisted transrectal manoeuvre, developed specifically for the left kidney in Jersey and Red Sindhi crossbreed cows, allows the left kidney to be explored in most animals [8]. However, fatty tissue, dehydration, low frequency transducers, and intestinal gas make US evaluation of the kidneys difficult [7].

Doppler US of the kidneys provides dynamic information about the renal blood flow associated with renal function [10] and about how this is modified by different physiological factors and/or pathological conditions [9,10,11,12,13,14,15,16,17,18,19,20]. It can be used as a guide for renal biopsies to reduce haemorrhage risk [7,21,22], by providing information about microvascular and parenchymal lesions [10]. 

In pulsed wave Doppler (PWD), characterization of arterial blood flow by the resistive index (RI) and the pulsatility index (PI) enables comparison of the velocity spectrum over time to evaluate the blood flow through the renal vascular bed. Both indices are also influenced by systemic parameters and postrenal factors such as heart rate, hepatic function, urinary drainage, blood pressure, glomerular filtration rate, and renal blood flow or anaemia [12,15]. However, previous studies have demonstrated that the main factors affecting the RI are renal tissue and vascular compliance [9,11,15]. 

Age, sex and body weight (BW) have been studied as potential factors influencing the RI in humans [11,19], dogs [20], cats [9,23], horses and donkeys [24,25]. Although sex has not been identified as an influential factor in any species, age [11,20] and BW can alter the RI [23]. Differences between right and left kidney RI have also been investigated in humans, dogs, horses, and cats, and although contradictory results have been reported [9,11,12,20,24], some difference can be observed in hypertensive patients or can be considered indicative of urinary obstruction [20] or a result of anatomical and physiological differences between the right and left kidney [25]. Other influencing factors such as breed and the effect of training have also been investigated in cats [26] and horses [24].

Both RI and PI can be altered even with normal or minimal changes in both the blood renal markers and in the B-mode US, indicating that these indexes are early markers in the disease process, e.g., in diabetic nephropathy [10,11,19]. In cattle, urea levels are not always correlated with kidney damage, because of protein and ruminal metabolism [6,7], and therefore a significant amount of tissue damage must occur before the values of the blood parameters increase. 

Resistive indexes values are considered valuable for evaluating diffuse acute or chronic kidney disease in cats [9,10] and dogs [15], and they can also be elevated in hydronephrosis, end-stage kidney disease and renal lithiasis. When B-mode US does not show any significant changes, RI thus serves as an early sign of altered kidney function [15], acting as a prognostic tool in chronic kidney disease and also enabling close monitoring [10]. Resistive indices are correlated with renal histological changes in dogs, cats and humans [10]. There is a notable lack of information regarding normal Doppler flow characteristics of the bovine kidney in the literature. Thus, the aims of the present study were to establish a protocol for renal Doppler US in adult healthy Holstein-Friesian cows and to provide reference values for renal RI and PI. Cut-off values are proposed on the basis of the results, to help clinicians and researchers distinguish normal and diseased kidney, as in humans, dogs and cats.

## 2. Materials and Methods 

All experiments were performed following Spanish standards for the protection of animals used for scientific purposes. The procedures applied were supervised by the Bioethics Committee of the Rof-Codina Veterinary Teaching Hospital, University of Santiago de Compostela (Spain) and met the criteria for non-invasive procedures (AELU001/19/ALT (08)/ANAT (01)/DBV/01, 14/11/2019).

### 2.1. Animals

Twenty-five healthy, non-lactating, non-pregnant adult Holstein-Friesian cows, of mean age 7.16 ± 1.01 years and mean weight 541 ± 48 kg, used for teaching clinical examination methods, were included in the study. The cows received a daily maintenance ration of hay (provided ad libitum) and 2 kg of concentrate (given in two equal rations at about 08:00 h and 19:00 h) throughout the study period. Values of haematological, biochemical and urine parameters were normal in all of the cows [27]. The cows were housed in the installations of the Rof-Codina Veterinary Teaching Hospital, University of Santiago de Compostela, Spain.

### 2.2. Anatomy

**Figure 1:**
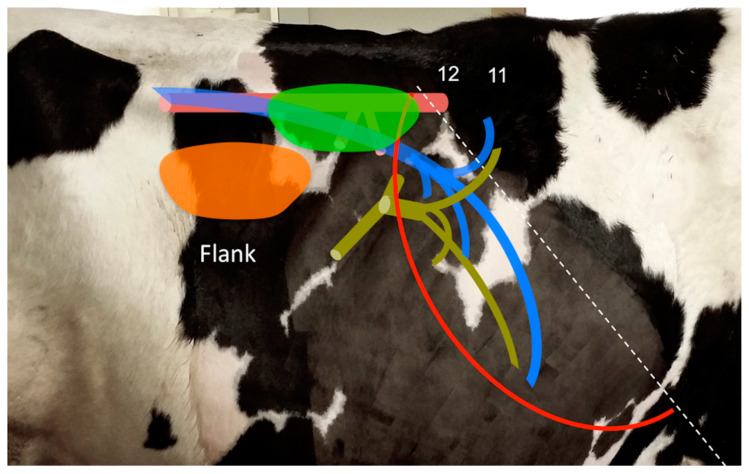
Anatomical location of the right kidney (bright green) and left kidney (orange) in the adult cow, as viewed from the right flank. The location of the caudal vena cava and its main hepatic veins (blue), the portal vein and its intrahepatic tributaries (pale green), the aorta and its main branches (pink), and the liver (red line) and diaphragm (dotted white line) are also indicated. Numbers denote intercostal spaces.

Both kidneys are retroperitoneal and therefore associated with the dorsal aspect of the abdominal cavity, with the right kidney markedly cranial relative to the left kidney. The kidneys are lobulated, surrounded by perirenal fat, with a well-developed capsule. Superficial lobulation corresponds to parenchymal division in renal lobules or pyramids. The right kidney is positioned at the level of the 12th intercostal space (ICS) and the 2nd or 3rd lumbar vertebra [7,28], of average length 18–25 cm (craniocaudal dimension), width 10–12 cm (lateromedial dimension) and thickness 5–6 cm (dorsoventral dimension) [7,29]. The cranial pole of the right kidney is in direct contact with the renal impression of the liver [7] (Figure 1).

The left kidney varies in position depending on ruminal distension and can be located from the midline to the right abdomen and rotated to a variable extent, sometimes even coming in contact with the right abdominal wall, normally more ventral than the right kidney [7]. Its dimensions are similar to those of the right kidney (19–25 cm, [7]), but 2–5 cm shorter for some authors [29], at the level of the 2nd to the 5th lumbar vertebrae. The left kidney is rotated 90° on its long axis, so that the hilum faces the dorsal aspect of the abdomen. The renal hilum is positioned relatively cranial in the renal parenchyma of both kidneys and is the site of entry and exit of the vessels and ureter. The renal sinus contains the primary and secondary segmental arteries and veins, the renal pelvis (dilated or not), two major calices (cranial and caudal), and a variable number of minor calices (13–64) [30]. These minor calices are in close contact with renal papillae from the apical aspect of renal pyramids [7]. In the periphery of the renal parenchyma, the renal pyramids are divided into cortex and medulla. Between the renal pyramids, the segmental arteries progress peripherally giving rise to smaller arcuate arteries that pass between the cortex and medulla.

One renal artery emerges from each kidney from both sides of the abdominal aorta, the left one slightly caudal and longer than the right renal artery. At the level of the renal hilum (just before or in the renal sinus), the main renal artery divides along the long axis of the organ into two to four primary segmental arteries, and each of these then divides into one to four secondary segmental arteries distributed throughout the renal parenchyma [22].

### 2.3. B-Mode and Doppler Ultrasound

As part of a general study of the normal abdominal ultrasound examination in adult cows, a transcutaneous abdominal ultrasound exploration of the right abdomen was performed, from caudal to cranial, as previously described [7,31]. The ultrasound examination was conducted while the animals were standing in stocks without sedation. All examinations were performed between 17:00 and 19:00, always by the same operator (J.D.B.-V.). The right abdominal skin was routinely prepared for ultrasonography, as previously described [31,32]. Alcohol and ultrasound gel were applied to optimize ultrasound probe contact with the animal´s skin. A complete B-mode, colour and pulsed wave Doppler (PWD) ultrasound examination of the kidney/s was performed with a low to mid-frequency convex array transducer (1–8 MHz) (MyLab-ClassC^®^, Esaote, Italy) and depth; frequency and gain were adjusted to optimize the ultrasound image at any time.

**Figure 2:**
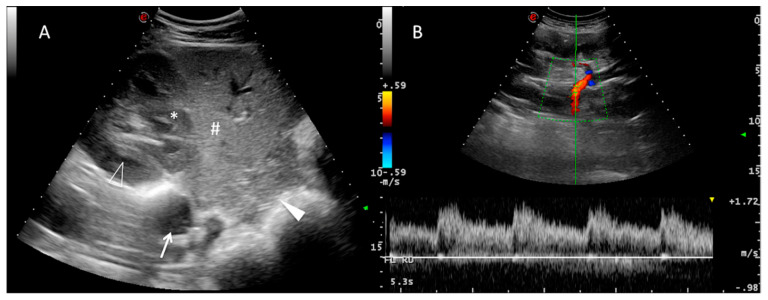
Ultrasound images of the right kidney. (**A**): Transverse B-mode image of the right kidney in close contact with the liver for comparison of echogenicity. The renal cortex (*) is hypoechoic relative to the hepatic parenchyma (#). The renal medulla of one renal pyramid (hollow arrowhead), caudal vena cava (arrow) and pancreas (solid arrowhead) are also highlighted in this image. (**B**): Longitudinal B-mode image where the colour-coded Doppler demarcates the renal hilum and the main renal artery entering the kidney. A spectral waveform from the renal artery is visible with pulsed wave Doppler, with four consecutive and similar cardiac cycles visible over time (X-axis). The arterial flow (shown in red) towards the transducer, i.e., towards the kidney, confirms that it is the artery and not the renal vein.

Transverse and longitudinal B-mode images of each accessible kidney were recorded to measure maximal craniocaudal, lateromedial and dorsoventral diameters (relative to the animal’s position), as well as to assess the overall echotexture and echogenicity in the right kidney, in comparison with the liver parenchyma (Figure 2A). 

Colour-coded Doppler ultrasound superimposed on the B-mode image was then applied to visualize the renal blood supply to identify which vessels were useful for PWD examination. In order to obtain a PWD spectrum, sample size (4–9 mm) and location were adjusted for the vessel of interest, with the aim of obtaining an angle of insonation always less than 60°, and as close to 0° as possible for optimal Doppler signal. Baseline, pulse repetition frequency (PRF) and gain were also adjusted in Doppler mode in order to optimize the Doppler trace, remove artefacts (e.g., aliasing) and improve blood flow measurements. At least three consecutive and similar cardiac cycle waveforms were used to consistently obtain resistive indices for each vessel, and at least three independent measurements were obtained for each accessible kidney (Figure 2B). These measurements were analyzed offline with specific software (MyLabDesk, Esaote, Italy), by tracing the maximum velocity spectrum from the beginning to the end of the cardiac cycle. Thus, the peak systolic velocity (PSV), end-diastolic velocity (EDV) and time average maximum velocity (TAMV) of the cardiac cycle were included and measured by the ultrasound software. RI values were calculated as [(PSV-EDV)/EDV] and PI values as [(PSV-EDV)/TAMV]. The ultrasound software automatically calculated these parameters. Proposed upper cut-off values were calculated on the basis of mean results, as described in the following section.

### 2.4. Statistical Analysis

All statistical tests were conducted using SPSS for Windows v.24 (IBM Corp., Armonk, NY, USA. The normality of the data distribution was checked using the Kolmogorov–Smirnov test. As the data were normally distributed, the results are presented as means and standard deviations. The cut-off values (upper limit) were calculated as:

(1) $$\bar{X_{j}}+1.96SD_{j}$$

where $\bar{X_{j}}$ is the mean of the variable *j* and $SD_{j}$ its standard deviation. One-way ANOVA was used to check for possible differences attributable to age, weight and right or left kidney. A significance level of *p* < 0.05 was applied in all cases.

## 3. Results

B-mode US of both kidneys (when visible) took between 5 to 10 min long. Doppler measurements were more difficult to obtain depending on individual factors such as cooperation, amount of intestinal gas, availability of the left kidney, and difficulties to locate and obtain quality Doppler measurements. Complete exploration time was not longer than 45 min in any case.

### 3.1. B-Mode US

None of the cows examined had any B-mode US abnormalities in the kidneys or adjacent structures, and none showed notable discomfort during the procedure. The mid-to-low frequency convex transducer (1–8 MHz) enabled good tissue penetration, with acceptable definition and Doppler signal in the kidneys visualized. The right kidney was visible in all animals, whereas the left kidney was only visible in 3 of the 25 animals. Access in both kidneys was from the right paralumbar fossa, craniodorsal for the right kidney (at the level of the cranial lumbar vertebrae, just below the transverse processes) and in the middle part for the left kidney, and slightly ventral relative to the right kidney (see Figure 1). Medulla and cortex were identified in all kidneys. The renal cortex was more echoic than the medulla, and iso- to hypoechoic relative to the hepatic parenchyma (Figure 2A). The left kidney cortical echogenicity was subjectively compared with the contralateral kidney and liver, and similar echogenicity was observed in both cases.

The B-mode dimensions of both kidneys are summarized in Table 1. The craniocaudal diameter of both kidneys was estimated because this dimension was larger than the maximal aperture of the probe image. 

### 3.2. Doppler US

All visible kidneys were accessible through the right paralumbar fossa, and Doppler measurements were made at the renal hilum. Transverse and longitudinal planes were useful for aligning sample size in the Doppler mode at the hilum of the right kidney because the renal artery was directed in a lateromedial direction. For the left kidney, a transverse plane was most useful because the renal hilum was positioned in an oblique dorsoventral direction. 

Doppler measurements of the right kidney were possible in 23 of the 25 animals examined. Doppler traces could not be obtained in two individuals because of a large amount of intestinal gas in the right abdomen that only could be used for comparison of echogenicity and renal dimensions. 

When evaluating the vessel architecture by means of colour Doppler US, the main renal artery and its main tributaries (segmental arteries) were easily visible (Figure 2B). The distal interlobar and arcuate arteries could not be successfully mapped because of their small size and respiratory motion. Thus, only the main renal artery at the renal hilum and segmental arteries were included in the study.

The Doppler waveform of the renal artery and its main tributaries was characterized in all animals as a low-resistance profile [11] with a slow decrease in velocity once the peak systolic velocity was reached (Figure 2B). A small spectral window was visible under the blood waveform. No double systolic peak was detected, although a broad systolic peak was usually present, with slight generalized turbulence of the Doppler profile. The results of RI and PI are presented in Table 2. Age and weight did not influence the RI and PI. No differences were found between right and left kidney indices. On the basis of these results and applying the previous described formula, the upper cut-off values for RI and PI were 0.63 and 1.00 respectively.

## 4. Discussion

To the best of our knowledge, this is the first report describing renal resistive indices in healthy, non-pregnant, non-lactating adult Holstein-Friesian cows. The findings serve as a basis for future studies comparing normal and diseased animals and/or different conditioning factors such as pregnancy and milking. Normal ranges of RI and PI are reflected, with proposed cut-off values of 0.63 and 1.00 for RI and PI respectively. The observed Doppler waveform corresponded to a low resistance profile with continuous diastolic flow, but with a small spectral window, indicating a higher velocity profile than more distal arteries [11]. Some turbulence was also detected in the Doppler profile of the renal artery and its main tributaries, which was partly explained by the large size of the vessel, which increased the Reynolds number, and some tortuosity extending from the aorta to the renal artery, as described for the portal system [31].

Both RI and PI are indirect measurements of blood flow resistance. The renal RI reflects changes in intrarenal perfusion but is also associated with systemic hemodynamics, and it has been suggested to provide useful prognostic information in human patients [18]. Renal RI is influenced by numerous hemodynamic and physiological factors such as vascular compliance, different pressures (pulse, urethra, intra-abdominal) and plasma renin activity [16]. The RI values obtained in the present study were within the range of reference values reported for healthy adult humans [11], dogs and cats [10,12], and horses and donkeys [24,25]. 

A cut-off value for renal resistive indices is a clinically useful tool for the diagnosis of healthy or pathological status. In the present study, we suggest an upper cut-off value for the RI of 0.63 and for the PI of 1.00 in healthy Holstein-Friesian cows. Different RI cut-off values have been established in human medicine, ranging from 0.58 [19] to 0.70 [11], partly because of variable normal values, which are affected by age and other physiological factors. Ostrowska et al. [17] consider 0.71 and 0.68 as the upper normal limits of RI in interlobar renal arteries in dogs and cats respectively; these values are similar to those suggested by Novellas et al. [12]. 

The limit may be more similar to that in other large animals, but there is no relevant information available in the scientific literature. Recent studies in horses and donkeys [24,33] did not establish a cut-off value, but the RI values were below 0.7 for both kidneys, as in our study. In relation to PI, the value we propose in cattle is much lower than those reported for dogs (1.52) and cats (1.29) [12]. The PI may be more accurate than the RI for differentiating abnormal waveforms because it also incorporates mean velocity as a parameter. Doppler measurements of the main renal artery and its main tributaries were made from within the renal hilum, located with the help of colour-coded Doppler US. Measurements at these locations provide general information about renal resistance, but they could be not as sensitive to reflect parenchymal changes as measurements made at the distal segmental arteries or arcuate arteries [11,17]. It was not possible for us to obtain a consistently good enough Doppler trace to measure RI and PI values at distal arteries due to the depth of examination and thickness of the skin (which requires low US frequency [7], with the subsequent lower detection of smaller vessels and insufficient Doppler signal), and, mainly, respiratory motion (that continuously moved the vessel of interest out of view). Nevertheless, some studies have demonstrated similar values from within or outside the renal parenchyma in humans [11], and in Persian cats [26], in which a RI value was reported for the interlobar arteries (0.52 ± 0.06) and also for the renal artery (0.54 ± 0.07) and aorta. We hypothesize that similar values would be obtained at more distal arteries, but further research must be performed with higher frequency transducers to confirm this.

Due to the anatomic branching of the renal artery [22], the ultrasound probe should preferably be aligned on a dorsal plane to produce the Doppler trace and make subsequent measurements. Nevertheless, in vivo ultrasound must always consider the angle of insonance in relation to the ability and the availability of good US approaches to the renal hilum. Thus, at the renal hilum, both transverse and dorsal planes were useful for obtaining the Doppler trace of the main renal artery and their primary tributaries. 

As previously pointed out, there was no apparent difference between the right and left kidney, and the values of the RI and PI indices were similar. However, this may have been due to the fact that the left kidney was rarely visualized (3/25) in our study, as previously observed [7]. As far we are aware, this is the first report describing normal RI values for cattle, and therefore, there is no information available in the scientific literature with which to compare our results. Our findings are consistent with those of other studies, in humans [34,35], in healthy dogs and cats [12,36], donkeys [24], and horses [33] that did not find any differences between the right and left kidney in physiological conditions, although other studies have reported differences in horses [24,25] and cats [9]. Although it is difficult to explain this difference, more data must be collected to determine whether the difference is due to technical or anatomical variants. A notable difference between the RI in the left and right kidney is considered an important diagnostic factor in detecting upper urinary tract obstructions in human patients [37]. Renal RI values are not specific markers of any particular type of kidney disease but help the clinician to detect renal impairment [10,33], even when subtle or no change in B-mode US is visible. 

It has previously been established that these indices are typically constant in adults, both in humans [11,18,19] and animals [20,24], as in the present study. Nevertheless, the values are different from those in new-born and childhood [9,17,20]. Siwinska et al. [33] also found that RI values were higher in elderly horses (>18 years) than in foals and adults, but were always below the upper value of 0.70. These differences should be investigated in future studies in cattle. 

Regarding the weight of the animal, most veterinary research has not found any correlation between renal RI and body weight, similarly to our study in cattle. Park et al. [38] described a weak correlation in cats, whereas Ostrowska et al. [17], Freccero et al. [24] and Siwinska et al. [33] did not detect any statistically significant differences in respectively cats/dogs or horses/donkeys.

Until now we have only considered healthy individuals, but these indexes can vary in pathological conditions or be affected by different factors such as gestation and/or lactation. For example, it has been reported that increased vascular resistance that could alter these parameters may be more common in dogs with kidney and liver diseases [14,15]. Endocrinopathies such as diabetes mellitus and hyperadrenocorticism [13,16] can modify renal RI, and correlations with hypertension and glucose levels are observed. Matos et al. [23] reported a cut-off value of 0.639 for cats suffering chronic kidney disease. In humans, Maksoud et al. [19] reported an upper limit of 0.58 in diabetics whereas in healthy people the limit is usually considered 0.70 [11].

None of the animals showed notable discomfort during the procedure, indicating that a standing exploration is a suitable approach for bovine abdominal ultrasound in daily practice. A low to mid-frequency convex array transducer (1–8 MHz) proved ideal because of its wide aperture and optimal combination of penetration and image quality. Although higher frequency and linear transducers provide better image resolution, they are only efficient in a superficial, narrower field of view, making them less useful for exploring the whole kidney parenchyma through a transabdominal window. Both cortex and medulla were clearly defined in our study, in contrast to the study of Seif and Bakr [4] in which no differentiation was observed in the right kidney through a transcutaneous approach. Technological advancements in US equipment in recent years have improved image quality even with low frequency transducers, combining good penetration and acceptable image quality, as in our study.

B-mode echogenicity, texture and kidney architecture were consistent with previous descriptions of normal kidneys, hypo to isoechoic between the renal cortex and the liver parenchyma [7]. The left kidney was only located through a transabdominal approach in a few of the cows examined (3/25), as previously reported and attributed to the large gas-filled intestine concealing the left kidney [7]. The cows were not fasted in our study; however, fasting could reduce gastrointestinal gas, as described in previous reports [4,8], potentially increasing the probability of reaching the left kidney. Imran and Sharma described an assisted technique that displaces the left kidney to the right abdominal wall through transrectal palpation, allowing transcutaneous US of the left kidney in fasted, medium-sized cows [8]. However, as this assisted technique is not routinely performed in daily practice, it was not applied in the present study. Rectal US enables use of a higher frequency transducer and thus provides better images [7,8]. However, rectal US examination was not performed in this study as only the caudal part of the left kidney is usually accessible through a rectal approach in large cows, and transabdominal US was considered an easier and more readily available method for evaluating both kidneys. However, future studies should explore this option, along with the assisted manoeuvre described by Imran and Sharma [8], to gain access by combining transrectal and transabdominal ultrasound, to validate Doppler measurements of the left kidney through a rectal, transcutaneous window.

In both right and left kidneys, the craniocaudal diameter was smaller than previously reported, regarding both anatomical [29] and US [1,2,7] measures. Special difficulty was experienced in obtaining the maximal length (craniocaudal dimension), which was longer than that of the transducer image, and therefore, a subjective estimate was made. Specific software for elongated ultrasound images should be applied to correctly measure kidney length, but this was not available in the ultrasound system used. Seif and Bakr [4] described normal sizes for the left and right kidneys in crossbreed cattle. The larger sizes obtained in the present study can be explained by breed selection. 

Although this is the first study that reports renal Doppler assessment in cattle, we are conscious that our study has some limitations. The inclusion criteria for the animals were normal physical examination with no history of renal disease, urinalysis, and serum creatinine and urea within the normal ranges, and no renal abnormalities in the B-mode US examination. In cattle, renal impairment can be a diagnostic challenge due to ruminal protein metabolism [7], and US can therefore help the clinician to detect kidney pathology. However, it is well known that diffuse pathology of the kidney often does not generate US changes [10,15], which is one limitation of our study. Nevertheless, the animals included in our study did not show any signs of chronic or acute pathology that affect the renal system. Another limitation is that blood pressure was not measured. Blood pressure was measured in other studies, in small animals [12], horses and donkeys [24,33], and humans [11]. Blood pressure has been described as an influencing factor in humans, but not in dogs [12] or horses and donkeys [24,33], and it can be ruled out as an influencing factor in cattle, in which hypertension is normally described as part of cardiovascular disease. Another limitation is that we considered the weight of the animals, but not the body condition score (BCS). Fatty tissue reduces the ability of the ultrasound beam to penetrate the tissues, and therefore, Doppler US images of these patients are expected to be of low quality, as with dehydrated patients [7,33]. Other possible influencing factors such as sex and breed should be considered, along with the previously mentioned factors, in future prospective studies.

## 5. Conclusions

Transabdominal US is a reliable and feasible tool for Doppler evaluation of the right kidney in cows, but only in the main renal artery and primary segmental arteries. Its availability for the left kidney is reduced but reliable when renal parenchyma is visible through the right paralumbar fossa. The technique is time consuming, but it is well tolerated by the animals. We suggest an upper cut-off value for the RI of 0.63 and for the PI of 1.00 in healthy non-pregnant, non-lactating Holstein-Friesian cows. Further studies are required in order to determine the relationship between renal indices and age (first stages of life), breed, sex, blood pressure, BCS, gestational influence, and milk yield, and to obtain reliable cut-off values for kidney pathology and other diseases. 

## Figures and Tables

**Table 1:** Sizes of right and left kidneys (in cm) measured by B-mode ultrasonography in adult healthy Holstein-Friesian cows. Data are means ± standard deviations.

Kidney	Dorsoventral Diameter	Lateromedial Diameter	Craniocaudal * Diameter
Right (*n* = 25)	6.64 ± 1.48	9.47 ± 1.82	17.04 ± 3.21
Left (*n* = 3)	11.51 ± 0.95	6.90 ± 1.90	17.61 ± 1.74

n: number of cows; * was estimated because this dimension was larger than the maximal aperture of the probe image.

**Table 2:** Resistive index (RI) and pulsatility index (PI) for the right and left kidneys in adult healthy Holstein-Friesian cows.

Variables	Right Kidney (*n* = 23)		Left Kidney (*n* = 3)	
	RI	PI	RI	PI
Mean	0.494	0.700	0.534	0.786
SD	0.069	0.145	0.051	0.110
Min	0.350	0.440	0.460	0.610
Max	0.670	1.110	0.610	0.970

SD: Standard deviation; Min: minimum value; Max: maximum value; n: number of cows.

## Data Availability

The data presented in this study are available on request from the corresponding author. The data are not publicly available due to privacy.

## References

[1] Braun U. (1991). Ultrasonographic examination of the right kidney in cows. Am. J. Vet. Res..

[2] Braun U. (1993). Ultrasonographic Examination of the Left Kidney, the Urinary Bladder, and the Urethra in Cows. J. Vet. Med. Ser. A.

[3] Hayashi H., Biller D.S., Rings D.M., Miyabayashi T. (1994). Ultrasonographic diagnosis of pyelonephritis in a cow. J. Am. Vet. Med. Assoc..

[4] Seif M.M., Bakr H.A. (2007). Ultrasonography of normal, cystic and dysplastic kidney in cattle. J. Vet. Med. Res..

[5] Floeck M. (2007). Sonographic application in the diagnosis of pyelonephritis in cattle. Vet. Radiol. Ultrasound.

[6] Braun U., Nuss K., Wehbrink D., Rauch S., Pospischil A. (2008). Clinical and ultrasonographic findings, diagnosis and treatment of pyelonephritis in 17 cows. Vet. J..

[7] Floeck M. (2009). Ultrasonography of Bovine Urinary Tract Disorders. Vet. Clin. N. Am. Food Anim. Pract..

[8] Imran S., Sharma S. (2014). Transcutaneous ultrasonographic examination of the left kidney in healthy cows. Vet. Med..

[9] Tipisca V., Murino C., Cortese L., Mennonna G., Auletta L., Vulpe V., Meomartino L. (2016). Resistive index for kidney evaluation in normal and diseased cats. J. Feline Med. Surg..

[10] Bragato N., Borges N.C., Fioravanti M.C.S. (2017). B-mode and Doppler ultrasound of chronic kidney disease in dogs and cats. Vet. Res. Commun..

[11] Scoutt L.M., Taylor K.J.W., Taylor K.J.W., Burs P.N., Wells P. (1999). El riñón. Aplicaciones Clínicas de la Ecografía Doppler.

[12] Novellas R., Espada Y., De Gopegui R.R. (2007). Doppler ultrasonographic estimation of renal and ocular resistive and pulsatility indices in normal dogs and cats. Vet. Radiol. Ultrasound.

[13] Novellas R., Ruiz De Gopegui R., Espada Y. (2008). Determination of renal vascular resistance in dogs with diabetes mellitus and hyperadrenocorticism. Vet. Rec..

[14] Novellas R., de Gopegui R.R., Espada Y. (2008). Increased renal vascular resistance in dogs with hepatic disease. Vet. J..

[15] Novellas R., Ruiz De Gopegui R., Espada Y. (2010). Assessment of renal vascular resistance and blood pressure in dogs and cats with renal disease. Vet. Rec..

[16] Chen H.Y., Lien Y.H., Huang H.P. (2016). Association of Renal Resistive Index, Renal Pulsatility Index, Systemic Hypertension, and Albuminuria with Survival in Dogs with Pituitary-Dependent Hyperadrenocorticism. Int. J. Endocrinol..

[17] Ostrowska J., Kiełbowicz Z., Zaleska-Dorobisz U., Atamaniuk W., Pietsch-Fulbiszewska A., Kinda W. (2016). Resistive index (RI) obtained in renal interlobar arteries of normal dogs and cats by means of Doppler ultrasonography. Pak. Vet. J..

[18] Di Nicolò P., Granata A. (2017). Renal Resistive Index: Not only kidney. Clin. Exp. Nephrol..

[19] Maksoud A.A.A., Sharara S.M., Nanda A., Khouzam R.N. (2019). The renal resistive index as a new complementary tool to predict microvascular diabetic complications in children and adolescents: A groundbreaking finding. Ann. Transl. Med..

[20] Agut A., Soler M., Fernández-Del Palacio M.J. (2020). Changes in Renal Resistive Index Values in Healthy Puppies during the First Months of Life. Animals.

[21] Mohamed T., Oikawa S. (2008). Efficacy and safety of ultrasound-guided percutaneous biopsy of the right kidney in cattle. J. Vet. Med. Sci..

[22] Szymanski J., Olewnik L., Wysiadecki G., Przygocka A., Polguj M., Topol M. (2018). Proposal for a new classification of the renal artery in the bovine kidney. Vet. Med..

[23] Matos I., Azevedo P., Carreira L.M. (2018). Pilot study to evaluate the potential use of the renal resistive index as a preliminary diagnostic tool for chronic kidney disease in cats. J. Feline Med. Surg..

[24] Freccero F., Petrucelli M., Cipone M., Nocera I., Sgorbini M. (2020). Doppler evaluation of renal resistivity index in healthy conscious horses and donkeys. PLoS ONE.

[25] Macrì F., Pugliese M., Di Pietro S., Coco M.A., Liotta L., Niutta P.P., Nardi S., Quartuccio M., Lanteri G., Palumbo Piccionello A. (2015). Doppler Ultrasonographic Estimation of Renal Resistive Index in Horse: Comparison Between Left and Right Kidneys. J. Equine Vet. Sci..

[26] Carvalho C.F., Chammas M.C. (2011). Normal Doppler velocimetry of renal vasculature in Persian cats. J. Feline Med. Surg..

[27] Kaneko J.J., Harvey J.J., Bruss M.L. (2008). Clinical Biochemistry of Domestic Animals.

[28] Budras K.D., Wünsche A., Budras K.D., Habel R.E., Mülling K.W., Greenough P.R., Wünsche A., Buda S. (2011). Lymphatic system, adrenal glands, and urinary organs. Bovine Anatomy. An Ilustrated Text.

[29] Sisson S., Sisson S., Grossman J.D., Getty R. (1982). Sistema urogenital de los rumiantes. Anatomía de Los Animales Domésticos.

[30] Pereira-Sampaio M.A., Bagetti Filho H.J.S., Carvalho F.S., Sampaio F.J.B., Henry R.W. (2010). A proposed new classification for the renal collecting system of cattle. Am. J. Vet. Res..

[31] Barreiro-Vázquez J.D., Miranda M., Barreiro-Vilanova M.I., Diéguez F.J., Barreiro-Lois A. (2019). Characterization of the Normal Portal and Hepatic Blood Flow of Adult Holstein-Friesian Cows. Animals.

[32] Barreiro-Vázquez J.D., Barreiro-Lois A., Miranda M. (2020). Ultrasonography of Normal Adrenal Glands in Adult Holstein–Friesian Cows: A Pilot Study. Animals.

[33] Siwinska N., Zak A., Slowikowska M., Szczepankiewicz B., Niedzwiedz A., Paslawska U. (2019). An assessment of the utility and repeatability of the renal resistive index in horses. PLoS ONE.

[34] Keogan M.T., Kliewer M.A., Hertzberg B.S., DeLong D.M., Tupler R.H., Carroll B.A. (1996). Renal resistive indexes: Variability in Doppler US measurement in a healthy population. Radiology.

[35] Ansarin K., Bavil A.S., Ghabili K., Shoja M.M., Khosroshahi H.T., Hajipour B., Tubbs R.S., Parvizi M. (2011). Are Doppler ultrasonography parameters symmetric between the right and left kidney?. Am. J. Clin. Hypn..

[36] Chang Y.J., Chan I.P., Cheng F.P., Wang W.S., Liu P.C., Lin S.L. (2010). Relationship between age, plasma renin activity, and renal resistive index in dogs. Vet. Radiol. Ultrasound.

[37] Sayani R., Ali M., Shazlee K., Hamid R.S., Hamid K. (2012). Functional evaluation of the urinary tract by duplex Doppler ultrasonography in patients with acute renal colic. Int. J. Nephrol. Renovasc. Dis..

[38] Park I.C., Lee H.S., Kim J.T., Nam S.J., Choi R., Oh K.S., Son C.H., Hyun C. (2008). Ultrasonographic evaluation of renal dimension and resistive index in clinically healthy Korean domestic short-hair cats. J. Vet. Sci..

